# A Text-book of Ophthalmology

**Published:** 1901-05

**Authors:** 


A TEXT-BOOK OF OPHTHALMOLOGY. By John C. Wright, A.M., M.D., Professor of Ophthalmology and Clinical Ophthalmology in the Ohio Medical University, etc. Second edition, with 117 illustrations. Published by P. Blakiston's Son & Co. Philadelphia.

This book was written for the medical student, and for him who desires only a superficial knowledge of Ophthalmology it will be found a very suitable guide. Each subject is discussed with brevity, yet withal clearly. The arrangement tends to simplicity, the writing is pleasant to follow, and the illustrations are sufficient in number, and, being culled from the books of well-known authors, are usually helpful to the text. A good glossary is added.

While praising the book generally we do not consider it faultless. The author's operation for cataract extraction will we think not come into general use. We find no word of the removal of the lens as a treatment in myopia; and we read that "a concave glass should never be prescribed without placing the eye under the influence of a mydriatic," which we consider to be frequently quite unnecessary. The section on glaucoma might have been much more instructive. Its etiology has not received its proper importance. On the whole, however, we consider the book a trustworthy guide so far as it goes and recommend it. It is very well put together from the point of view of the publisher. A. W. S.

